# Collapsing focal segmental glomerulosclerosis in a patient with oral cavity cancer: A case report

**DOI:** 10.1097/MD.0000000000025857

**Published:** 2021-05-07

**Authors:** Sae Byeol Choi, Kyoung Min Kim, Moon Hyang Park, Kyung Pyo Kang

**Affiliations:** a Department of Internal Medicine, Research Institute of Clinical Medicine, Jeonbuk National University Medical School; b Biomedical Research Institute, Jeonbuk National University Hospital; c Department of Pathology, Jeonbuk National University Medical School, Jeonju, Korea; d Department of Pathology, Konyang University Hospital, Daejeon, Republic of Korea.

**Keywords:** collapsing variant, focal segmental nephrotic syndrome, nephrotic syndrome, oral cavity cancer

## Abstract

**Rationale:**

Focal segmental glomerulosclerosis (FSGS) is one of the most common glomerular diseases, leading to end-stage renal disease. Among the 5 variants of FSGS, the collapsing variant is rare and has the worst prognosis. Solid and hematologic malignancies are associated with glomerular diseases, such as membranous nephropathy, minimal change disease, and FSGS. However, squamous cell carcinoma of the oral cavity is rarely associated with nephrotic syndrome, especially FSGS.

**Patient concerns:**

A 55-year-old woman diagnosed with oral cavity cancer presented with generalized edema with heavy proteinuria and renal dysfunction after neoadjuvant chemotherapy and wide surgical excision.

**Diagnosis:**

Renal biopsy shows segmental or global collapse of glomerular capillaries with marked hyperplasia and swelling of overlying epithelial cells, suggesting a collapsing variant of FSGS.

**Interventions:**

After the renal biopsy, we prescribed oral prednisolone at a dose of 1 mg/kg/day. Despite immunosuppressive treatment, renal function deteriorated, and hemodialysis was started.

**Outcomes:**

After 23 sessions of hemodialysis and high-dose oral glucocorticoid treatment, renal function gradually improved, and oral glucocorticoid therapy was discontinued after 8 months. Currently, this patient is in a cancer-free state and has normal renal function without proteinuria.

**Lessons:**

Unusual collapsing FSGS might be associated with neoadjuvant chemotherapy and wide surgical excision in patients with oral cavity cancer. Proper diagnostic workup, such as renal biopsy and high-dose glucocorticoid therapy, might have helped recover from nephrotic syndrome and acute renal injury in cancer patients.

## 1 Introduction

Focal segmental glomerulosclerosis (FSGS) is one of the most common glomerular diseases, leading to end-stage renal disease. Among the 5 variants of FSGS, the collapsing variant is rare and has the worst prognosis. Clinically, collapsing FSGS is associated with black racial predominance and human immunodeficiency virus-associated nephropathy. It has also been associated with various conditions, such as viral infection (parvovirus B19) and drugs (pamidronate, interferons, and anthracycline). However, most cases are idiopathic.^[1,2]^

Solid tumor-associated membranous nephropathy (MN) and Hodgkin lymphoma-associated minimal change disease (MCD) are well-known paraneoplastic glomerulonephritis.^[3]^ MCD occurs in approximately 1% of Hodgkin's lymphoma, and FSGS also occurs in approximately 0.1% of cases.^[3]^ However, squamous cell carcinoma of the oral cavity is rarely associated with nephrotic syndrome, especially FSGS. Here, we present a case of collapsing FSGS in a patient with oral cavity cancer who presented with heavy proteinuria, edema, and acute kidney injury after neoadjuvant chemotherapy and surgery. We successfully treated temporary hemodialysis and high-dose glucocorticoid treatment after confirmation of FSGS by renal biopsy.

## 2 Ethical approval

Ethical approval was received from the Jeonbuk National University Hospital Institutional Review Board (CUH 2019-12-027). Written informed consent for publication was obtained from the patient.

## 3 Case presentation

**Figure 1:**
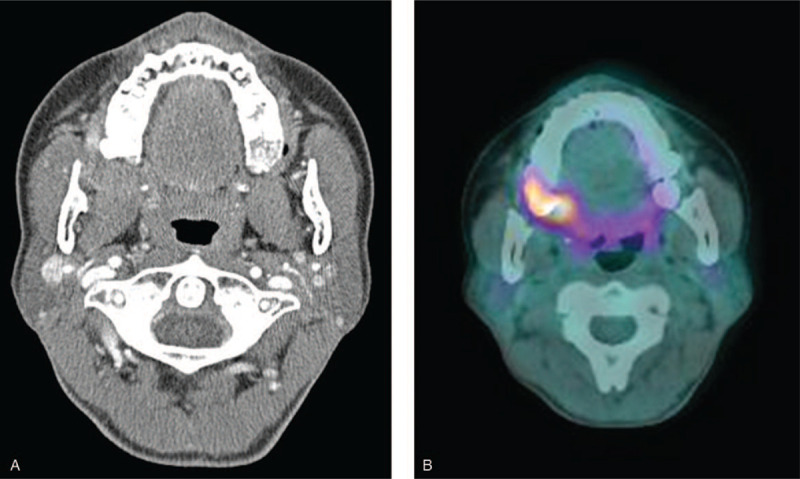
A) Spiral CT of the neck. There is a 2.4 × 1.6 × 2.0 cm sized enhanced mass around the right retromolar trigon. The mass is extended to the right buccal space and invades into part of the pterygoid muscle. B) Whole-body FDG PET/CT. There is FDG-avid mass lesion (SUVmas = 8.58) around the right retromolar trigon. CT = computed tomography, FDG = focal segmental glomerulosclerosis, PET = positron emission tomography.

A 55-year-old woman was consulted to evaluate renal dysfunction from the department of head and neck surgery. Two months ago, oral cavity cancer, histologically squamous cell carcinoma, was diagnosed. A 2.4 × 1.6 × 2.0 cm sized enhanced mass around the right retromolar trigon in neck computed tomography (Fig. 1A). The mass extended to the right buccal space and invaded part of the pterygoid muscle. Focal segmental glomerulosclerosis positron emission tomography/computed tomography showed FDG-avid mass lesion around the right retromolar trigon (Fig. 1B). When diagnosing oral cavity cancer, the renal function was normal, except for 1+ proteinuria on urinalysis. Surgeons decided to be treated with 1 cycle of neoadjuvant chemotherapy (docetaxel and cisplatin) and wide tumor excision.

**Figure 2:**
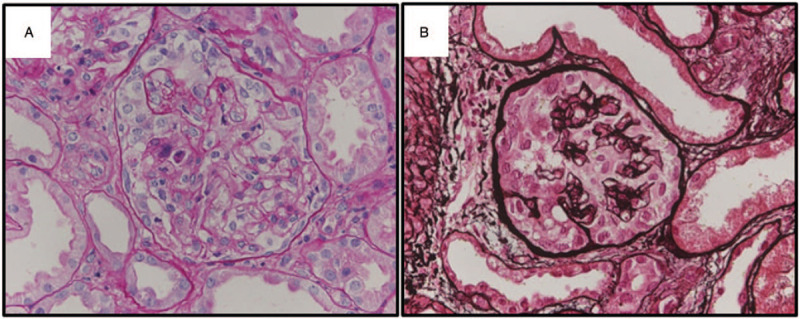
Renal biopsy findings. A and B) Representative glomerulus showing the segmental or global collapse of capillaries due to marked hyperplasia and swelling of overlying epithelial cells. Glomerular capillary lumina contain lymphocytes (PAS and Methenamine silver stain, x400).

One month after neoadjuvant chemotherapy and surgery, generalized edema with heavy proteinuria and severe renal dysfunction developed. On physical examination, a coarse breathing sound with rhonchi was heard in both lower lung fields, and 2+ pretibial pitting edema on both lower extremities was noted. Laboratory findings revealed a white blood cell count of 7090/mm^3^, hemoglobin level of 9.9 g/dL, platelet count of 287,000/mm^3^, blood urea nitrogen level of 58 mg/dL, serum creatinine level of 3.67 mg/dL, and serum albumin level of 2.7 g/dL. Urinalysis revealed 4+ proteinuria with hematuria. The urine protein/creatinine ratio was 17578.51 mg/g. Viral markers for hepatitis B, C, and human immunodeficiency virus were negative. Anti-nuclear antibody, anti-neutrophil cytoplasmic antibody, and C3/C4 levels were within normal limits. We performed the ultrasound-guided renal biopsy. The findings showed segmental or global collapse of glomerular capillaries with marked hyperplasia and swelling of overlying epithelial cells, suggesting a collapsing variant of FSGS (Fig. 2). There is patchy mononuclear inflammatory cell infiltration in the interstitium and mild interstitial fibrosis with tubular loss and atrophy. Electron microscopic findings showed diffuse effacement of the foot process of the external capillary surface, segmental wrinkling of the glomerular basement membrane, and no immune deposits in the mesangium.

**Figure 3:**
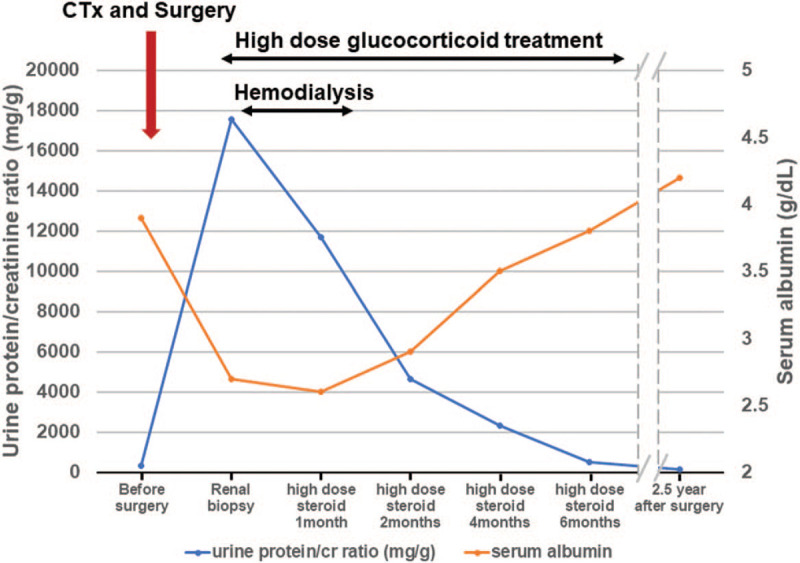
The clinical course after diagnosis of oral cavity cancer. (CTx, neoadjuvant chemotherapy with docetaxel and cisplatin).

Initially, we prescribed oral prednisolone at a dose of 1 mg/kg/day. Despite immunosuppressive treatment, renal function has deteriorated. We performed hemodialysis after internal jugular vein catheterization. After 23 sessions of hemodialysis and high-dose oral glucocorticoid treatment, renal function slowly recovered to a serum creatinine level of 1.6 mg/dL and started to taper the glucocorticoid. Eight months after initial treatment, we discontinued oral prednisolone and checked urinalysis and renal function regularly. After 2 and half years of follow-up, the patient was in a cancer-free state and maintained normal renal function (Fig. 3).

## 4 Discussion

Collapsing FSGS is a rapidly progressive form of FSGS characterized by unusual histologic features of focal segmental and global collapse of the glomerular capillary walls with marked visceral cell hyperplasia.^[4]^ Clinically, the patient had heavy proteinuria, resistance to glucocorticoid treatment, and a rapid course of renal failure.^[5]^ Glomerular diseases are common in solid or hematologic malignancies with a paraneoplastic process or the use of a chemotherapeutic agent. The most common form of glomerular disease is MN, with a prevalence of 10% to 22% in biopsy-proven MN.^[6–8]^ Although MN is the most commonly described glomerular disease associated with solid tumors, MCD or FSGS rarely occurs. FSGS is usually associated with renal cell carcinoma and thymoma, with few cases reported in lung, breast, and esophageal cancers.^[9]^

Some drugs are associated with glomerular diseases. Bisphosphonates are associated with podocyte injuries such as MCD or FSGS, particularly collapsing FSGS.^[5]^ Anthracycline chemotherapeutic agents such as adriamycin and daunomycin have also been associated with collapsing FSGS.^[10]^ These drugs are toxic to podocytes and lead to extensive podocyte loss and subsequent repopulation of parietal epithelial cells.^[5]^ Multiple mechanisms are involved in podocyte loss or injury via apoptosis, detachment, or failure to proliferate, which results in podocytopenia and glomerulosclerosis.^[11]^ Podocyte apoptosis results from elevated transforming growth factor-β, angiotensin II, reactive oxygen species, and reduced levels of cyclin-dependent kinase inhibitors p21 and p27.^[12]^ Podocyte detachment is critically related to α3β1 integrin or dystrolglycan in the glomerular basement membrane.^[13,14]^ A decreasing number of podocytes is a consequence of a lack of appropriate proliferation after podocyte injury.^[15]^

General measures for the treatment of collapsing FSGS include blood pressure control, minimization of proteinuria by angiotensin inhibition, and lipid-lowering therapy.^[5,16]^ The standard treatment was an immunosuppressive agent such as a high oral glucocorticoid for at least 8 to 16 weeks. Alternatively, calcineurin inhibitors or alkylating agents with or without low-dose glucocorticoid regimens can also be used in cases of steroid-related adverse events or steroid-sensitive and steroid-resistant FSGS.^[5]^ In addition to immunosuppressive treatment, treatment of underlying conditions such as infection, drugs, or tumors can lead to remission.

One retrospective study showed that collapsing FSGS, compared with not otherwise specified FSGS, is associated with severe nephrotic syndrome with renal impairment but has similar renal survival after appropriate immunosuppressive treatment.^[17]^ Therefore, early diagnosis and initiation of proper immunosuppressive therapy are essential in patients with collapsing FSGS.

Our patient received neoadjuvant chemotherapy for the treatment of oral cavity cancer. However, we prescribed docetaxel and cisplatin, which are toxic to tubules, not podocytes. Before starting neoadjuvant chemotherapy, the patient had 1+ proteinuria on urinalysis. Therefore, we postulated that this case might have developed full-blown nephrotic syndrome after neoadjuvant chemotherapy and wide excision. This case showed an excellent response to an 8-month high-dose glucocorticoid treatment, which was unusual in collapsing FSGS.

In conclusion, this case shows an unusual collapsing FSGS in a patient with oral cavity cancer after neoadjuvant chemotherapy and wide excision. It has been successfully treated with emergency hemodialysis and high-dose glucocorticoid treatment after renal biopsy. Proper diagnostic workup such as renal biopsy for heavy proteinuria with renal dysfunction in a patient with oral cavity cancer and timely immunosuppressive therapy might have preserved renal dysfunction.

## Author contributions

**Conceptualization:** Kyung Pyo Kang.

**Data curation:** Sae Byeol Choi, Kyoung Min Kim.

**Formal analysis:** Kyoung Min Kim, Moon Hyang Park, Kyung Pyo Kang.

**Funding acquisition:** Kyung Pyo Kang.

**Supervision:** Moon Hyang Park, Kyung Pyo Kang.

**Validation:** Kyoung Min Kim, Moon Hyang Park, Kyung Pyo Kang.

**Writing – original draft:** Sae Byeol Choi.

**Writing – review & editing:** Kyung Pyo Kang.
